# How to succeed in implementing community-based breeding programs: Lessons from the field in Eastern and Southern Africa

**DOI:** 10.3389/fgene.2023.1119024

**Published:** 2023-03-20

**Authors:** Aynalem Haile, Tesfaye Getachew, Mourad Rekik, Ayele Abebe, Zelalem Abate, Addisu Jimma, Joram M. Mwacharo, Joaquin Mueller, Berhanu Belay, Dawit Solomon, Emil Hyera, Athumani S. Nguluma, Timothy Gondwe, Barbara Rischkowsky

**Affiliations:** ^1^ International Centre for Agricultural Research in the Dry Areas, Addis Ababa, Ethiopia; ^2^ Debre Berhan Agricultural Research Center, Debre Berhan, Ethiopia; ^3^ Bonga Agricultural Research Center, Bonga, Ethiopia; ^4^ Areka Agricultural Research Center, Areka, Ethiopia; ^5^ National Institute for Agricultural Technology (INTA), Bariloche, Argentina; ^6^ International Livestock Research Institute (ILRI), Addis Ababa, Ethiopia; ^7^ Tanzania Livestock Research Institute, West Kilimanjaro Centre, Kilimanjaro, Tanzania; ^8^ Department of Animal Sciences, Lilongwe University of Agriculture and Natural Resources, Lilongwe, Malawi

**Keywords:** breeding schemes, lessons learnt, innovative approaches, sustainability, Africa

## Abstract

Breeding programs involving either centralized nucleus schemes and/or importation of exotic germplasm for crossbreeding were not successful and sustainable in most Africa countries. Community-based breeding programs (CBBPs) are now suggested as alternatives that aim to improve local breeds and concurrently conserve them. Community-based breeding program is unique in that it involves the different actors from the initial phase of design up until implementation of the programs, gives farmers the knowledge, skills and support they need to continue making improvements long into the future and is suitable for low input systems. In Ethiopia, we piloted CBBPs in sheep and goats, and the results show that they are technically feasible to implement, generate genetic gains in breeding goal traits and result in socio-economic impact. In Malawi, CBBPs were piloted in local goats, and results showed substantial gain in production traits of growth and carcass yields. CBBPs are currently being integrated into goat pass-on programs in few NGOs and is out-scaled to local pig production. Impressive results have also been generated from pilot CBBPs in Tanzania. From experiential monitoring and learning, their success depends on the following: 1) identification of the right beneficiaries; 2) clear framework for dissemination of improved genetics and an up/out scaling strategy; 3) institutional arrangements including establishment of breeders’ cooperatives to support functionality and sustainability; 4) capacity development of the different actors on animal husbandry, breeding practices, breeding value estimation and sound financial management; 5) easy to use mobile applications for data collection and management; 6) long-term technical support mainly in data management, analysis and feedback of estimated breeding values from committed and accessible technical staff; 7) complementary services including disease prevention and control, proper feeding, and market linkages for improved genotypes and non-selected counterparts; 8) a system for certification of breeding rams/bucks to ensure quality control; 9) periodic program evaluation and impact assessment; and 10) flexibility in the implementation of the programs. Lessons relating to technical, institutional, community dynamics and the innovative approaches followed are discussed.

## 1 Introduction

The failure of centralized nucleus breeding schemes and crossbreeding programs for small ruminants has called for a mindset shift for sustainable options of genetic improvement in low input systems. Recently, a more participatory approach started gaining global interest (Mueller et al., 2015). Called “community-based breeding,” it combines farmer training to improve selection methods, pooling community flocks to create a larger gene pool from which breeding animals can be selected, technical support to provide farmers with information on breeding options, data collection and analysis to evaluate individual animal performance. This approach is inherently sustainable as it engages the communities, hence supports local-level decision making, focuses on locally adapted indigenous breeds, considers the constraints that smallholder farmers face and empowers farmers’ organizations (cooperatives) in low input systems.

Genetic improvement of livestock is often viewed as a complex process that requires technical and organizational sophistication. In Europe, animal breeding has been traditionally supported by the State where large national breeding programs have been implemented. Currently, these programs are mostly run and financed by farmer cooperatives/breeds’ associations and include data recording and processing, and the evaluation of the genetic merit of individual animals. In developing countries, the appropriate infrastructure to implement such programs is largely unavailable. Therefore, past attempts to replicate developed-country approaches often mismatched goals and targets, could not fit into low-input systems with many producers each owning small flock sizes, and have resulted in little success.

Community-based breeding programs cover a range of situations (e.g., Sölkner et al., 1998; ICAR-FAO, 2000; Haile et al., 2018) but typically target low input systems and farmers within limited geographical boundaries having a common interest to work together to preserve and improve their genetic resources (Mueller et al., 2015). They focus on indigenous stock and consider farmers’ needs, views, decisions and active participation, from inception through to implementation, and therefore provide a participatory and bottom-up approach. Their success is based upon proper consideration of farmers’ breeding objectives, infrastructure, participation, and ownership (Sölkner et al., 1998; Wurzinger et al., 2011; Mueller et al., 2015; Haile et al., 2020). In low input small holder production systems, flock sizes are typically small, and this makes the design of conventional breeding programs difficult and there is a danger of inbreeding. Pooling flocks together, which is done in community-based breeding programs (CBBPs), helps avert the challenge.

In 2009, the approach was introduced to Ethiopia by the International Center for Agricultural Research in the Dry Areas (ICARDA) in partnership with the International Livestock Research Institute (ILRI), Austria’s University of Natural Resources and Life Sciences (BOKU), and the Ethiopian National Agricultural Research System. In Ethiopia, the implementation of CBBPs started with 4 communities representing different breeds and productions systems. These pilot CBBPs have since expanded to include more than 130 communities. Though implemented at a pilot scale in Ethiopia, the CBBPs have resulted in quantifiable genetic gains and impacted the livelihoods of rural communities (Haile et al., 2020). There are also on-going breeding programs for local goats of Malawi and Tanzania which have generated similar gains in goats from four and three communities, respectively (Kaumbata et al., 2020). The approach has also been introduced to other countries including, Burkina Faso, Iran, Liberia, South Africa, Sudan, and Uganda. Currently, CBBPs focusing on local genotypes are being advocated as the strategy of choice for genetic improvement of sheep and goats (Sölkner et al., 1998; Kosgey and Okeyo, 2007; Mueller et al., 2015; Haile et al., 2019, 2020).

Designing a CBBP is much more comprehensive than simply applying genetic theories to achieve increased productivity. Its implementation combines infrastructure, capacity development of national partners, community development, and the opportunity to improve farmer livelihoods by creating integrated processes for productive breeding of adapted animals and the markets for their products. By working with local breeds, CBBPs offer a framework to achieving goals of breed improvement and conserving the animal genetic resources. Several studies have been conducted to design suitable CBBPs for smallholder farming systems in Ethiopia, Malawi and Tanzania (Gizaw et al., 2009; Haile et al., 2018; Kaumbata et al., 2020).

These pilot schemes need to be scaled out to have significant impact on the lives of larger populations. For this to happen, the substantial knowledge and experience gained in these pilots and the lessons learnt, need to be communicated and shared to guide new CBBPs and sustain existing programs. In this paper the essentials for success of CBBPs, lessons learned and innovations by communities are highlighted. The knowledge gaps which need to be addressed are also identified with specific knowledge users in mind.

## 2 How community-based breeding programs were implemented in the Ethiopian, Malawi and Tanzania pilots

CBBPs combine selection of breeding rams/bucks based on systematic recording of important flock productivity improvement parameters, such as body weight at 4–6 months and lambing/kidding interval, with expert local opinion as to what constitutes a good ram/buck and communal use of selected rams/bucks. Farmers who wish to participate are organized into sheep/goat breeding associations, many of which evolve into formal cooperatives with a prominent financial profile (Haile et al., 2018). Local enumerators are recruited to help with data collection, which is then managed in a database and analyzed by scientists from local research centers to inform selection decisions. Extension staff are involved and they are educated on the required technical aspects to facilitate successful implementation of CBBPs.

All animals in a community are treated as one flock and two stages of ram/buck selection are usually applied: initial screening when traditionally sales of young lambs/kids occur (at 4–6 months of age) and final selection of yearling for admission to breeding. All young rams/bucks are collected at a central location in each community on an agreed screening date. Selection is then carried out based on the estimated breeding values or on selection indexes constructed to improve agreed multi-trait breeding objectives.

A breeding ram/buck selection committee comprised of 3–5 members that are elected by the community is involved in the selection. If, for example, 15 rams/bucks were to be selected from 100 candidates, 20 would be pre-selected based on their breeding values and the committee will then rank the selected rams/bucks and cull the last five. To arrive at the decision, the committee examines the conformation, coat colour, presence or absence of horns, horn type, tail type and other criteria. The number of rams/bucks to be selected depends on the number of ewes/does available for mating while accounting for the replacement rates. Unselected rams/bucks can be castrated, fattened and marketed for meat production. Ram/buck rotation can be practiced in order to avoid inbreeding as these rams/bucks can only stay and be used for breeding in the community for a maximum of 3 years and should be culled once its daughters are ready to be mated. The culled rams/bucks if still young can be sold as a breeding animal to other communities. In Ethiopia, the pilot CBBPs have been designed and implemented since 2009 by a team of researchers from ICARDA, ILRI, BOKU University, Austria and Ethiopian National Agricultural Research Centers. The pilot CBBPs were supported through various projects funded by multiple donors. The day-to-day follow-up of these CBBPs was done by the research and extension departments of the Ethiopian government. For Tanzania, the field implementation was supported by the government of Tanzania through the Tanzania Livestock Research Institute (TALIRI) and the local government authority of the respective districts where the program was implemented. For Malawi, pilot implementation by researchers from Lilongwe University of Agriculture and Natural Resources (LUANAR) started in 2015 with support from USDA, and backstopped by BOKU University. Department of Animal Health and Livestock Development and Department of Agriculture Research Services of Malawi collaborated in the project implementation.

## 3 Results from community-based breeding programs in Ethiopia, Malawi and Tanzania

In Ethiopia, there are more than 130 CBBPs with around 100 households each. As CBBP is a relatively new strategy for genetic improvement of small ruminants, the last 10 years have been spent on testing the functionality of the strategy and we have been refining and customizing the program to different species (sheep or goats), breeds, agro-ecologies and production systems. In Tanzania, we have started with 3 pilot CBBPs containing between 30 and 40 indigenous goat keeping households each. In Malawi, four CBBPs were established in 2013 with financial support by USDA. We have evaluated the biological and socio-economic performance of CBBPs in Ethiopia, Malawi and Tanzania and below are the findings as reported in Haile et al. (2020) and Kaumbata et al. (2020).

• Sheep/goat farming, once a side activity for the farmers in these countries, is now the main business and the linchpin of their livelihoods.
• High demand for breeding males from neighboring communities, other government programs and NGOs in all sites, provides the foundation for specific business models around production of breeding sires and semen for artificial insemination.
• In Ethiopia, more than 13,000 households in 130 villages derive direct benefits from the scheme and the emergence of a functional cooperative society in each village.
• Most of the participating households in Menz (a CBBP site in Ethiopia) have graduated from the government-run safety net program that meets short-term food needs through emergency relief. They now use income from the sale of sheep to meet their subsistence needs.
• “Best of stock” growing breeding lambs/kids, that were previously sold and slaughtered (“negative selection”), are now retained as breeding stock in all communities.
• Increased income from sheep and goat production (an average increase of 20 percent since CBBP inception in 2009 in Ethiopia) and increased mutton consumption (now an average of 3 sheep slaughtered for home consumption per family per year compared to 1 sheep at the start of the project) directly linked to CBBP production in Bonga, Horro and Menz sites in Ethiopia.
• Sheep/goats in CBBPs have shown improved performance, such as lamb/kid growth rate, lambing/kidding interval, reduced mortality and attract higher market prices compared to sheep/goats from non-CBBP farmers in all communities.
• Most of the established cooperatives have managed to build capital (e.g., Boka-Shuta cooperative in Ethiopia has about USD 110,000).

## 4 Lessons learnt from implementing community-based breeding programs

### 4.1 Technical

*Breeding objective definition*: there are many tools which can help define breeding objectives of communities, including structured surveys, choice card experiments, group and individual rankings (Duguma et al., 2011), bio-economic analyses or combinations of different approaches. However, given the complexity, resource need and the ultimate output generated, individual rankings offer the best option. This is very easy and allows the full participation of owners in choosing their best and worst animals from their flocks (Mirkena, 2010; Getachew et al., 2020).

*Community-based breeding program structures*: CBBPs should be tailored to different production systems. For instance, pastoral production systems need different schemes to mixed crop livestock systems (Getachew et al., 2022). In pastoral areas, the schemes must consider mobility patterns, larger flock sizes, and climate patterns leading to breeding objectives focusing on adaptive traits, etc. Communities with large flock sizes should be treated differently to those with small flock sizes. In the latter, households can pool their animals and selection can be organized from many flocks. However, in situations where individual household flock size is large, within flock (household) selection can be designed. Where some households keep large flocks, it may be difficult to identify and record all animals. In such cases, elite herds can be selected to serve as sires of dams based on interest of herd owners and individual animal performance. Other specific situations such as where flocks mix in communal grazing areas or where sires are separated from the flocks for various reasons, need to be evaluated as these would entail different sire use strategies.

*Performance and pedigree recording*: implementation of CBBPs should be simplified at the beginning. Selection of sires could start from simple mass selection where indexes could be constructed for maximum of three traits based on individual animal performance. This would be followed by calculating breeding values using spreadsheets (e.g., excel), after correcting for known variations. When experience is developed, selection can be based on estimated breeding values. BLUP breeding values are usually calculated considering the sire as “unknown” and therefore breeding values (BV) calculated with larger error variances and genetic trend will be underestimated. Henderson (1988) showed that by identifying possible sires and assigning to each a mating probability, one could estimate BV with greater accuracy. In many CBBPs pedigree databases, sire identification is uncertain rather than completely unknown. Farmers may be requested to provide possible sires with a mating probability estimation enabling the use of Henderson’s method to calculate BVs. The general lesson is that, inaction rather than the absence of perfect data is the major constraint in livestock breeding (Rege et al., 2011).

Performance and pedigree data recording is feasible in CBBPs (Gizaw et al., 2014). However, the characteristics and limitations of low input systems need to be considered. The general advice is, keep it simple and sustainable; agree on few/key economically important traits, especially at the start and align recording to routine practices (weaning, vaccination, sales, etc.).

Enumerators are very crucial for data collection and day to day follow-up of the breeding programs. Also, the extension is influential in facilitating the implementation of these programs. The extension staff are responsible for the provision of extension services and, they play a critical role of linking farmers with researchers (Kaumbata et al., 2020). Furthermore, public support is crucial for sustainability of the breeding programs. Governments should invest on some of the complementary services and hire enumerators over a longer period until the community becomes economically viable to absorb their costs.

*Capacity development* of the different actors, mainly farmers is extremely important for the success of CBBPs. Farmers need to be trained on basic animal husbandry, including healthcare, proper feeding, and selection practices. Cooperative leaders could also be trained on leadership, financial management and bookkeeping. Tailored trainings need to be organized for different actors in CBBP. Local researchers must be trained on implementation of CBBPs; focusing on data collection, management and analysis, animal ranking and sire use and mating plans; reproductive management and application of reproductive biotechnologies; flock health monitoring and health certification of the improved sires. Breeding programs need long-term commitment and support from different actors. Technical support from research and extension partners mainly in data management, analysis and feedback of estimated breeding values is crucial.

### 4.2 Institutional

Establishment of breeders’ cooperatives with clear by-laws and formal organizational structures are crucial for success of CBBPs. Although not uniform in all CBBP sites, groups of committees manage the cooperatives. These include, a main committee with a chair, a procurement committee, a control committee, a credit and savings committee and a capacity building committee. The committees are responsible for effective functioning of the breeding cooperatives and roles and responsibilities are shared among the committees. Overall, CBBP operation is managed by the cooperatives. Formally registered cooperatives are governed by their by-laws and members abide by their rules. Legally registered cooperatives had better management and financial resources, better selection and management of breeding rams (Gutu et al., 2015). The governments are keen to organize farmers and to support cooperatives. Formally registered cooperatives have access to free auditing services, training and support for financial record-keeping from district cooperative promotion offices.

Proper organizational link among the different actors in CBBP is crucial. In CBBP, as indicated earlier, there are cooperative committees at community level; team of researchers with team leader at research sites; and the CGIAR team. The day-to-day follow-up of CBBP including data collection is done by enumerators. The research team follows the activities on the ground including compilation of data collected by enumerators and estimation of breeding values and assist in selection decision. The research team also liaises with the implementing institutions on technical and financial matters.

These structures are very useful for close follow up and sustainability of the CBBPs. The close interaction also helps develop trust among the partners for similar interventions. The injection of revolving funds from projects, could help the cooperatives to purchase young sires that can be used for breeding. It also means that if a member needs cash, they could sell their young animal to the cooperative before selection decision is made so that the best breeding animals are retained in the community.

### 4.3 Community issues

Like any enterprise, communities need to see benefits from CBBPs for them to fully engage. Therefore, it is important that such schemes are properly planned with real benefits to farmers. Within-breed selection schemes will result in genetic improvement, improved productivity and profitability if properly executed (Haile et al., 2020). However, it should be noted that short-to perhaps medium-term returns on investment will most likely come from non-genetic gains, such as improvement in feeding, disease control and better reproductive management (for example, making breeding sires available in the required number to serve all females will result in more lambs/kids) and market linkages. Implementation of the CBBPs is also contributing to managing crosscutting issues including environmental conservation in the face of climate change mitigation and gender equity (Kaumbata et al., 2020). Therefore, genetic improvement effort should be part of an overall livestock development agenda across the whole value chain.

## 5 Innovations by the communities

CBBPs are implemented through clearly defined guidelines (Haile et al., 2018). However, in implementing CBBPs, communities innovate and do things differently and efficiently to strengthen their operations. Some examples of innovative approaches followed by communities in different CBBPs are summarized below.

• As indicated earlier, some of the cooperatives have built capital through sale of breeding sires and culled animals. This capital is being used for different purposes including uplifting the financial status of the members and others. They have therefore devised a system where they advance credit to their members and other cooperatives (https://bit.ly/2PpG4Xr).
• In Bonga (Ethiopia) CBBPs, the cooperative members agreed and are in the process of forming a breeding nucleus for elite ewes. They knew that not all ewes are of the same genetic merit and have started identifying best ewes based on their own criteria and will only allow breeding rams produced from these elite ewes to be used in the communities. The elite ewes shall be retained by their respective owner farmers and will not move into a central station. They are discussing mechanisms to reward farmers whose ewes are selected. Although the initiative is from the farmers, the research team will support the establishment of the nucleus with performance records derived from the breeding database. Hence, farmers selection criteria will be augmented with known performance data. Selection on the dam side has been found to result in genetic gain in CBBPs (Jembere et al., 2019), therefore, the breeder cooperatives are moving towards more effective selection.
• The cooperative leaders have established sub-groups based on neighborhoods and any information from both the research team and extension is channeled through the sub-groups to all the members and this ensures easy and reliable information flow and action.
• Ram/buck sharing and management has been one of the challenges in CBBPs. However, once bought by the cooperatives, the communities have developed different systems of sharing males and management of the potential candidate males. For example, in Bonga (Ethiopia), following the purchase of potential candidate rams, the cooperative leaders decide who keeps the ram depending on the number required in the mating group, individual experience in managing rams etc. The farmer manages the communal rams for the period the ram is in service, and thereafter when the ram is sold the profit realized from its sale (i.e., the difference between the cost when the young ram was bought and when sold) is shared between the farmers and the cooperative. Similar management of bucks was adopted in Malawi CBBP sites.
• Close follow-up is an important element for a successful CBBP. This is done by the research and extension team. However, in one of the sites (Bonga, Ethiopia) the cooperative leaders also took initiative to supervise their members every month and provide feedback to the research center, enumerators and their members.
• In the Abergelle (Ethiopia) goat CBBP, each CBBP participant operates a savings bank account. All members unanimously agreed to save ETB 200 (equivalent to US$ 5) for every buck kid sold. This has cultivated a saving culture in the community.
• Integration of CBBPs into local or community leadership systems. In the beginning it is hard to have every farmer in the community to accept the idea, something which might be a hinderance in progress of the program. Some farmers may not be willing to cull their poorly performing animals and use those selected by the committee. In the communities in Tanzania through the involvement of village leaders, rules for successful CBBPs are set and agreed in village meetings and are reinforced locally.

## 6 Success factors

Based on the experience and lessons learnt from the implementation of CBBP pilots in the region, critical factors for the success of CBBPs were identified.

1. Identification of the right beneficiary following a clear guideline on who should be a member. Some essential factors to consider in selecting target communities for a CBBP as detailed in Haile et al. (2018) and include: a) External factors (market access, potential negative and/or positive impacts by other projects, synergies with other projects, government support, NGO support and availability of inputs and services); and b) community-related factors (willingness to participate in the program, prioritizing the species of interest, existence of communal/shared resources and/or institutional arrangements, presence of community leaders (elders) and champion farmers/pastoralists who are critical in socio-cultural structures in the region).
2. Institutional arrangements including the establishment of breeders’ cooperatives to support functionality and sustainability of the programs. There must be clear working modalities and implementation structures among the different CBBP actors, as detailed in Section 4.2. Legal cooperatives with clearly defined by-laws must be established for each CBBP.
3. Capacity development of the different actors on basic animal husbandry, breeding practices, estimation of breeding values and financial management. Capacity development of the different actors is of utmost importance for the success of CBBPs. The breeding programme should be supported by comprehensive extension work to train the farmers and boost their experiences and skills in small ruminant production techniques (Yapi-Gnaore, 2000). During that period, farmers should be informed of the long-term benefits they could derive from breeding programs and activities such as performance recording. Too little investment in expertise has contributed to low efficiency and in some cases failure of breeding programs and absence of science-based genetic improvement practices (Gizaw et al., 2018).
4. Breeding programs cannot be implemented without performance and pedigree recording. A mobile application for data recording and management would allow accurate recording and ease the job of the enumerators. Given the challenge of internet connection in villages of developing countries, an offline mobile application for data collection and management is vital. The International Center for Agricultural Research in the Dry Areas, in partnership with AbacusBio (https://abacusbio.com/), has established a cloud-based digital genetic database and data capture platform (DTREO) for Ethiopia, Tanzania and India. The platform captures and stores data and is designed for offline data capture in situations where internet connectivity is poor. Such a data system could be used.
5. Framework for dissemination of improved genetics and up/out scaling strategy. For CBBPs to have significant impact they need to scale. Improved genetics produced in CBBPs need to reach the production/base population. This requires a clear design as suggested by Mueller et al. (2019).
6. The expansion of a delivery system based on service provision in reproductive technologies such as artificial insemination (AI) to support the up/out scaling strategy, diet improvement at critical stages of the reproductive cycle and ultrasound-based pregnancy diagnosis mobile units to serve selection of the females for AI and to down-control infertility by identifying and culling problematic females.
7. Support for long periods by committed technical staff mainly in data management, analysis and feedback of estimated breeding values. We have clearly seen over the years that CBBPs that are supported and implemented by committed research and extension staff are the ones that succeed. While CBBP is a low investment intervention, it needs very close follow-up for the community to take up the challenge of ultimately running the programs.
8. Pro poor livestock development needs to consider the whole value chain development. This includes support in complementary services such as disease prevention and control, feeding interventions, market linkages for meat and breeding animals.
9. To ensure quality control, a system for certification of improved rams/bucks by an authorized body is needed. The quality and value of selected sires is the backbone of breeding programs. The vision is to gradually move from producing genetically improved sires to establishing a reliable stud where breeding excellence is certified. Emerging breeding programs are hindered and can collapse prematurely when farmers cannot access superior males of good breeding quality, reproductive and health standard.
10. Evaluation of the program and assessment of impact of the scheme. An integral component of a functional CBBP is monitoring technical and management issues related to the implementation of the breeding program; whether outputs, outcomes and impacts are achieved or achievable; and whether mechanisms to ensure sustainability of the breeding program are in place.

Table 1 summarizes the major requirements for setting up CBBPs and the support services that are needed. It also highlights the available knowledge, what needs to be done and the suggested institution to lead it.

**TABLE 1:** Major requirements for setting up community-based breeding programs and support services needed.

Components of the community-based breeding program	Existing knowledge	Knowledge we have generated	Knowledge gaps in the existing interventions	Who are the potential institutions/organizations (national/subnational) to be engaged in designing/implementing the actions
Definition of breeding objectives and selection traits	Tools to undertake interviews, choice experiments, group ranking experiments	Own flock ranking experiment (Duguma et al., 2010)	Rapid method to determine initial selection traits to be followed by more comprehensive approach to breeding objectives	Lead: Ethiopia Livestock Development Institute (ELDI); Tanzania Livestock Research Institute (TALIRI); Lilongwe University of Agriculture and Natural Resources (LUANAR)
				Site level: Ethiopia Regional and Federal Agricultural Research Institutes (ERFARI); Tanzania Regional Administration and Local Government Authorities (TRALGA); Local and International NGOs that promote livestock livelihood projects
Breeding structures for different systems	Centralized nucleus breeding structures	Community-based breeding structures (Mirkena et al., 2012; Haile et al., 2018; Jembere et al., 2019; Getachew et al., 2020)	Refining breeding structures for pastoral production systems with large flock sizes	Lead: ELDI; TALIRI; LUANAR
				Site level: ERFARI; TRALGA; NGOs
Data recording and management system	Development of database for data recording has been a challenge. Many efforts did not succeed	Dtreo, data recording and management flatform (https://dtreo.io/)	Inbuilt system for estimation of breeding values and ranking of sires	Lead: ERFARI; TALIRI; LUANAR
Dissemination of improved genetics	Distribution of improved sires to the base population	Methodological framework for optimized dissemination of improved genetics (Mueller et al., 2019)	Field testing of the framework is going on; to have the dissemination program conceptualized and implemented by the communities	Lead: Ethiopia Ministry of Agriculture, extension division; TALIRI; Malawi, Department of Animal Health and Livestock Development (DAHLD)
				Site level: District level livestock bureau, local enumerators and extension staff
Reproductive biotechnology as a tool for dissemination of improved genetics	Seasonality and rhythms of reproduction of indigenous sheep and goat breeds in their homelands	Response to potential synchronization protocols (Rekik et al., 2016); Validation of a simple, cost-effective oestrous synchronization protocol—Organization and functioning of low-infrastructure artificial insemination mobile laboratories (Besufkad et al., 2020)	Easy methods for cooling semen to reach distant communities	ERFARI; TALIRI; Tanzania National Artificial Insemination Center (NAIC)
			Synchronizing artificial insemination data with the core breeding program data	ELDI; TALIRI; LUANAR
			Certification of breeding sires based on genetic merit, reproductive potential and health status	Regional animal production and animal health divisions
Breeders cooperative establishment	No formal association	Legal breeders cooperative with clear by-laws (Gutu et al., 2015)	Build their capacity; access to rural micro-financing	National regulations: Ethiopia Ministry of Agriculture, cooperative office; Tanzania Cooperative Development Commission (TCDC); Malawi Ministry of Trade and Industry
				Site level: District Office of cooperatives
Institutionalization of the breeding program	Centralized breeding programs run by government	Breeding programs run by community through legal breeders cooperatives, supported by NARS and the extension division (Haile et al., 2018, 2020)	Experiences with the pilot schemes taken to scale; Strengthening of breeders cooperatives to make them a viable commercial enterprise	Lead: ELDI; TALIRI; LUANAR
				Site level: ERFARI; TRALGA, Local enumerators and extension staff in Ethiopia, Tanzania and Malawi
Markets for breeding and meat animals	Informal markets which are inefficient	Evidence generated on the benefit of market facilities and market information system (Kassie et al., 2020) to marketing of small ruminants; evidence on policy induced price distortions (Kassie et al., 2019)	Marketing models tailored to different goat and sheep markets	Regulations: Ministry of agriculture/Livestock, marketing department; Site level: Stakeholder communities of practice under development
Evaluation of breeding programs	No formal comprehensive evaluation framework available in Ethiopia	Framework and evidence on both biological and socioeconomic evaluation of CBBPs (Haile et al., 2018, 2019; 2020; Lamuno et al., 2018)	Incorporation of the evaluation framework in the national breeding programs	Lead: ELDI; TALIRI; LUANAR
				Site level: Ethiopia regional and federal agricultural research institutes; TALIRI zonal centers; LUANAR CBBP sites

## 7 Conclusion

Community-based breeding program is a new approach that has stimulated global interest. It has been implemented in Ethiopia since 2009 and scaled to Malawi and Tanzania as an alternative to the often-unsuccessful centralized nucleus breeding programs. Different schemes were designed and implemented in different production systems in the countries. The results indicated that measurable genetic gain could be achieved for important breeding goal traits and CBBPs resulted in socio-economic benefit to the communities. For the success of such schemes, we have identified factors that need to be followed. Additionally, there are several lessons drawn from these schemes and, innovative approaches were also followed by some communities which either solved emerging problems or helped to ensure sustainability of such schemes.

## Data Availability

The original contributions presented in the study are included in the article/supplementary material, further inquiries can be directed to the corresponding author.
